# E-tool for mental health coping: usability and effectiveness study of a biofeedback approach on a digitized diaphragmatic breathing strategy

**DOI:** 10.1192/j.eurpsy.2023.1822

**Published:** 2023-07-19

**Authors:** R. Maçorano, F. Canais, A. Perdigão, I. Gonçalves, M. Parreira, M. Ribas, H. A. Ferreira

**Affiliations:** 1 Faculty of Sciences of the University of Lisbon; 2 Instituto Superior Técnico, Lisboa; 3 NeuroGime, Braga, Portugal

## Abstract

**Introduction:**

One of the most common somatic responses of the human body to a mental health issue consists of alterations of the breathing rate. Typically, when an individual is under stress, tends to have a more rapid shallow breathing - instead of resorting to the diaphragm to help the air in and out the lungs, ends up conducting a thoracic breathing, leading to extended fatigue or dizziness.

**Objectives:**

The aim of this project is to assess the accuracy and efficacy of measuring the breathing rate through abdominal breathing movements, via the smartphone’s sensors, and applying it to personalize a digitized diaphragmatic breathing strategy. The main hypothesis under testing is that the digitization of this strategy with the personalization to the subject’s own response is efficient as a valuable tool for mental health coping.

**Methods:**

A tool was developed and integrated with a mobile app that aggregates mental health coping strategies, based on the digitalization of positive psychology techniques. The tool included the diaphragmatic breathing exercise and the personalization to the user through biofeedback. Such biofeedback was based on the user’s abdominal movements and directly impacted the course of the strategy. The tool is under testing, counting so far with 25 subjects resident in Portugal.

**Results:**

The usability and effectiveness metrics of the solution will be assessed on the first contact of the subject with the app, and segmented by different subject profiles. Mental health metrics will also be assessed, namely anxiety levels - using the smartphone sensors and standard psychiatric scales. The results will be compared with a control group, in which the subjects will only perform the self-assessment, without using the breathing exercise.

**Image:**

**Figure:**
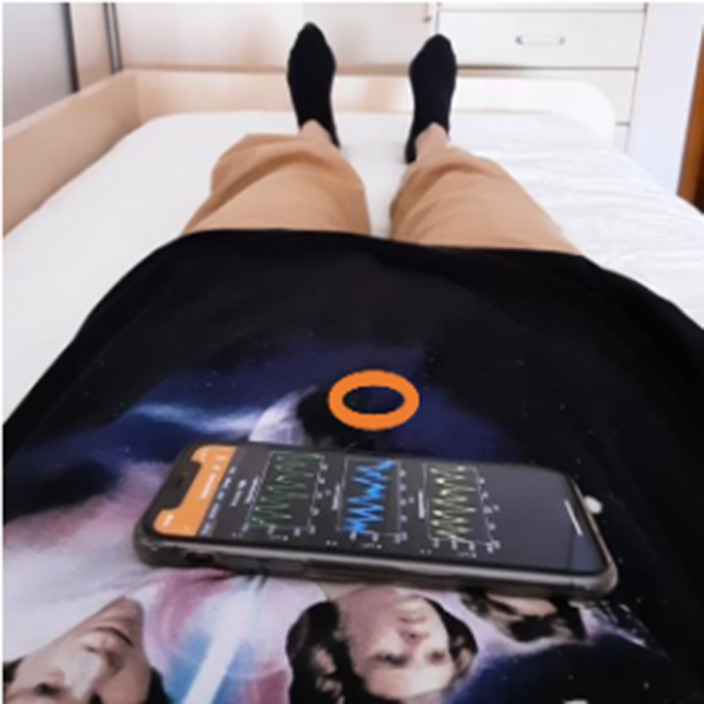

**Conclusions:**

We have yet to draw conclusions from the project; however, we aim to achieve the first results in due time.

**Disclosure of Interest:**

None Declared

